# Autistic Adults are Not Impaired at Maintaining or Switching Between Counterfactual and Factual Worlds: An ERP Study

**DOI:** 10.1007/s10803-021-04939-4

**Published:** 2021-03-11

**Authors:** Heather J. Ferguson, Lena Wimmer, Jo Black, Mahsa Barzy, David Williams

**Affiliations:** School of Psychology, Keynes College, University of Kent, Canterbury, Kent CT2 7NP England, UK

**Keywords:** Autism, Language comprehension, Counterfactuals, Event-related potentials, N400

## Abstract

We report an event-related brain potential (ERP) experiment that tests whether autistic adults are able to maintain and switch between counterfactual and factual worlds. Participants (N = 48) read scenarios that set up a factual or counterfactual scenario, then either maintained the counterfactual world or switched back to the factual world. When the context maintained the world, participants showed appropriate detection of the inconsistent critical word. In contrast, when participants had to switch from a counterfactual to factual world, they initially experienced interference from the counterfactual context, then favoured the factual interpretation of events. None of these effects were modulated by group, despite group-level impairments in Theory of Mind and cognitive flexibility among the autistic adults. These results demonstrate that autistic adults can appropriately use complex contextual cues to maintain and/or update mental representations of counterfactual and factual events.

Counterfactual statements depict hypothetical events that are counter to reality. Such utterances are used in day-to-day communication to express emotions such as regret (‘if only I had done that…’) or relief (‘at least I didn’t do that…’), and their use is important in generating alternative actions that could have led to better outcomes. Counterfactuals thus have an important role in learning from mistakes and planning for future behaviour and decision making (Coricelli & Rustichini, 2010; Epstude & Roese, 2008; Zeelenberg & Pieters, 2007).

Despite their frequent use in everyday communication, comprehending counterfactual statements involves sophisticated cognitive processes. Understanding a counterfactual statement, such as “If Sue had checked the weather forecast, she would have brought her umbrella”, requires the listener to comprehend the imagined, hypothetical events (that Sue checked the weather forecast and brought her umbrella), to infer the implied factual events (that Sue didn’t check the weather forecast, she didn’t bring an umbrella, and it is raining), and to understand that only the latter is true in reality (Byrne, 2002; Fauconnier, 1994). A growing body of empirical evidence supports this dual mental models account of counterfactual processing, with some online measures revealing cognitive costs associated with processing counterfactual statements relative to factual statements (e.g. de Vega et al., 2007; Ferguson, 2012; Ferguson & Sanford, 2008; review: Kulakova & Nieuwland, 2016). These studies have largely examined counterfactual processing within isolated narratives (e.g. Black et al., 2018; Ferguson et al., 2019; Nieuwland & Martin, 2012; Nieuwland, 2013), which may not reflect the way that counterfactuals are used in everyday discourse, since interlocutors move rapidly between factual and counterfactual alternatives in natural dialogue. The importance of tracking the availability of factual/counterfactual events over longer narratives is highlighted in a series of studies conducted by de Vega and colleagues. They used self-paced reading and probe word tasks to show that that both initial (factual) and new (counterfactual) information were equally accessible immediately following a counterfactual context, but the counterfactual events become less accessible than factual after a short delay (de Vega et al., 2007; Urrutia et al., 2012). In the current paper, we aimed to systematically test the relative availability of factual and counterfactual models of the world, using event-related brain potentials (ERPs) to assess whether readers can switch between different models of the world during comprehension.

Previous research has explored this question using a reading anomaly detection task and observing modulations on the N400 ERP component (Ferguson & Cane, 2015). The N400 response is a negative-going ERP wave that peaks approximately 400 ms after a critical word onset, and has been shown to be a robust and sensitive measure of contextual integration and predictability in linguistic processing (Kutas & Federmeier, 2011). In two experiments reported in Ferguson and Cane (2015), participants read an initial sentence, which set up a counterfactual scenario (e.g. “If it had rained this morning, Susan would have rushed to get to work”), followed by a second sentence that manipulated linguistic cues to either maintain the counterfactual world (“In the end, Susan would have arrived at work early”) or switch back to the factual world (“In the end, Susan arrived at work late”). Passages depicting a factual context (e.g. “Because it had rained this morning, Susan had rushed to get to work”) were included as a baseline measure of contextual integration. ERPs were time-locked to a critical word in the second sentence (underlined in the examples), that was either consistent or inconsistent with the sentence context. Results revealed a more negative-going N400 response for inconsistent *versus* consistent critical words when the counterfactual context was maintained in the second sentence, suggesting that the counterfactual premise was preferentially used for semantic integration. In contrast, no consistency effect was found when readers were required to switch to the implied factual world in the second sentence, which suggests that both versions of the world were equally available to readers at the point of integration, and that readers had not yet switched to favour the factual interpretation of events. Interestingly, the speed with which readers detected contextually inconsistent input was influenced by individual differences in cognitive resources; individuals with high working memory capacity were faster and more reliable at detecting inconsistent words in a counterfactual context. In the current paper, we adapt this paradigm to test the availability of factual and counterfactual information in a group of autistic adults,^1^ who experience deficits in both social processing and executive functioning.

Autism Spectrum Disorder (ASD) is a pervasive developmental disorder associated with difficulties in social communication, and restricted interests and repetitive behaviours (American Psychiatric Association, 2013). On a cognitive level, autistic people (including those who are intellectually high-functioning, with IQ scores > 70) show group-level difficulties with executive functioning compared to age- and IQ-matched neurotypical peers (e.g. working memory and cognitive flexibility; Habib et al., 2019; Williams & Jarrold, 2013), and may be impaired at updating mental representations of language (Peleg et al., 2018). These atypical processing styles in autism have traditionally been attributed to general difficulties integrating information in context (known as ‘weak central coherence’, WCC; Booth & Happé, 2010; Frith, 1989; Martin, & McDonald, 2003), since autistic people tend to show a local-rather than global-focused processing style (Frith, 1989; Frith & Happé, 1994; Happé & Frith, 2006; but see Van der Hallen et al., 2015). More recently, researchers have proposed that autistic individuals have impaired meta-learning abilities, which disrupt their ability to contextualise incoming information and make predictions based on experience (known as the ‘predictive coding theory of autism’; Van Boxtel & Lu, 2013; Van de Cruys et al., 2014). Both of these accounts would predict that online counterfactual understanding is disrupted in autism.

On one hand, research on counterfactual thinking in children is consistent with accounts that have proposed impaired contextual access in autism. Autistic children experience difficulty distinguishing reality from fiction (e.g. Surian et al., 1996), perform less well on counterfactual reasoning tasks (Grant et al., 2004; Leevers & Harris, 2000), and deploy distinct strategies when producing counterfactual alternatives (Begeer et al., 2009), compared to their typically-developing (TD) peers. However, more recent empirical research with autistic adults has shown that in fact, real-time counterfactual understanding is undiminished, or even enhanced, in autistic adults compared to TD adults (Black et al., 2018, 2019; Ferguson et al., 2019). These studies used eye-tracking to measure the ease with which participants integrated events in short factual or counterfactual narratives, and varied the demands they placed on readers’ executive capacities, imagination, and emotional reasoning. Results showed that autistic adults were sensitive to anomalies within counterfactual narratives, and elicited appropriate anomaly detection responses (longer reading times and increased regressions) in a comparable or even faster timecourse than a matched TD group. This shows that autistic adults do not struggle with counterfactual thinking per se, though it is possible that difficulties might arise when the demands on cognitive load are increased. This possibility is compatible with the complex information processing theory which posits that the cognitive profile in autism reflects a general deficit integrating information across distributed cortical systems and using top-down knowledge when task demands are high (CIP; Minshew et al., 1997, 2008; Minshew & Goldstein, 1998; Williams et al., 2015).

It is important to note that Black et al. (2018) and Ferguson et al. (2019) established counterfactual worlds in single sentences that maintained a single world representation. Achieving coherence of information within these short utterances may be less challenging than comprehending the sort of counterfactuals involved in everyday discourse. In natural dialogue, speakers switch frequently between counterfactual and factual alternatives, which increases demands on contextual integration, working memory and cognitive flexibility. Thus, it is possible that autistic adults experience difficulties in processing when these demands are higher. The two-statement counterfactual utterances in Ferguson and Cane (2015), especially the condition that requires readers to switch from a counterfactual to factual world, provide a more naturalistic test of counterfactual comprehension. Accordingly, they may present a disproportional challenge for autistic adults, given the aforementioned difficulties with executive functioning and context integration in narrative comprehension.

The current study addresses these questions by replicating the ERP reading anomaly detection task in Ferguson and Cane (2015), comparing autistic and TD adults. We expected to replicate the general patterns seen in Ferguson and Cane (2015), with a context by consistency interaction revealing a consistency effect on the N400 when passages maintained a counterfactual or factual context, but not when readers were required to switch from a counterfactual to factual world. More importantly, we tested the prediction that these effects would be modulated by group. Specifically, we expected autistic participants to show appropriate N400 responses when either the factual or counterfactual world was maintained across the two sentences, but tested whether the onset of these contextual anomaly detection responses might be delayed in the autistic group compared to the TD group (by testing effects in early and late windows of the N400 component). In addition, given group-level difficulties with cognitive flexibility in autism, we expected that autistic participants would show a reversed consistency effect to critical words when the context required a switch from the counterfactual to the implied factual world, as readers continued to process words according to constraints from the counterfactual world.

## Method

### Participants

Twenty-five autistic adults (16 males) and 24 age-, sex-, and IQ-matched TD adults took part in the experiment (see Table 1), all of whom gave written, informed consent before participating. One autistic participant was excluded from analyses due to excessive noise in ERP data, leaving 24 participants in each group. This sample size is comparable to or exceeds the sample sizes used in previous research that has examined language comprehension in autistic and TD adults (e.g. Au-Yeung et al., 2015, 2018; Black et al., 2018, 2019; Ferguson et al., 2019; Howard et al., 2017), and the total sample exceeds that used in Ferguson and Cane (2015; N = 30). A power analysis using the Shiny app for performing 'exact' simulations of factorial experimental designs (http://shiny.ieis.tue.nl/anova_exact/) showed that this sample size yields an estimated power of 77.3% to detect a significant 3-way interaction between group, context and consistency with a medium effect size of *f* = 0.32 and significance level of α = 0.05.

**Table 1:** Demographic information for autistic and TD groups (M (SD)), showing between group t-tests (where *p < .05, **p < .01 and ***p < .001)

	Autistic (*n* = 24)	TD (*n* = 24)	*t*	*p*	*Cohen’s d*
Sex (m:f)	16:8	16:8			
Age (years)	32.78 (11.14)	34.00 (11.16)	0.38	.708	0.11
Performance IQ	103 (22.84)	107 (9.92)	0.71	.484	0.23
Verbal IQ	104 (12.46)	103 (11.71)	− 0.29	.776	0.08
Full-scale IQ	104 (17.33)	106 (10.22)	0.45	.657	0.14
Total AQ	31.33 (7.61)	18.83 (5.31)	− 6.58	< .001***	1.91
ADOS-2 module 4	8.25 (4.89)	–			
Animations task	4.37 (2.41)	5.80 (1.70)	2.22	.032*	0.68
WCST perseverative errors	14.29 (12.36)	5.54 (4.25)	− 3.28	.002**	0.95

IQ was assessed in all participants using the Wechsler Abbreviated Scale of Intelligence (WASI, Wechsler, 1999). Autistic participants had all received formal diagnoses of autistic disorder (*n* = 12), or Asperger’s Syndrome (*n* = 12), according to DSM-IV/5 or ICD-10 criteria (American Psychiatric Association, 2013; World Health Organization, 1993). Diagnostic reports were verified by the researchers. Current ASD features were assessed by a trained research-reliable assessor among participants in the autistic group using the Autism Diagnostic Observation Schedule-Generic (ADOS, Lord et al., 2000).

All participants completed the Autism-spectrum Quotient (AQ; Baron-Cohen et al., 2001), a self-report questionnaire that assesses ASD/ASD-like features. Mean scores for the AQ in each group are shown in Table 1. All participants were over the age of 18, were native English speakers, had normal or corrected to normal vision, and did not have a diagnosis of dyslexia or intellectual disability. Participants in the TD group did not report any current psychiatric diagnoses. The experiment was approved by the School of Psychology Research Ethics Committee, University of Kent.

### Materials and Design

One hundred and eighty experimental items were taken from Ferguson and Cane (2015; Experiment 2), each consisting of two sentences. The full set of experimental items are available on the Open Science Framework (OSF) website (see https://osf.io/ms5cy/). The first sentence presented a scenario in either a factual (e.g. “Because Karl had been wearing a jacket, he hadn’t minded the long delay.”) or a counterfactual context (e.g. “If Karl had been wearing a jacket, he wouldn’t have minded the long delay.”). The second sentence described a consequence of this event, which either referenced events to the factual world (e.g. “After waiting outside for an hour he now felt…”), or included a modal inflection that signalled a continuation of events according to the counterfactual world (e.g. “After waiting outside for an hour he would have felt…”). Crucially, this second sentence contained a critical word that was either consistent or inconsistent with the preceding context (e.g. “warm” *vs.* “cold”; see Table 2 for full example). This resulted in a within-subjects design that crossed three levels of context (factual *vs.* counterfactual-counterfactual *vs.* counterfactual-factual) with two levels of consistency (consistent *vs.* inconsistent).

**Table 2:** An example experimental item shown in each of the six conditions of the reading anomaly detection task

Factual	Consistent	Because David had been wearing his glasses, he was able to read the poster easily. From this distance, David found that the words were clear on the poster
	Inconsistent	Because David had been wearing his glasses, he was able to read the poster easily. From this distance, David found that the words were blurry on the poster
Counterfactual–counterfactual	Consistent	If David had been wearing his glasses, he would have read the poster easily. From this distance, David would have found that the words were clear on the poster
	Inconsistent	If David had been wearing his glasses, he would have read the poster easily. From this distance, David would have found that the words were blurry on the poster
Counterfactual–factual	Consistent	If David had been wearing his glasses, he would have read the poster easily. From this distance, David found that the words were blurry on the poster
	Inconsistent	If David had been wearing his glasses, he would have read the poster easily. From this distance, David found that the words were clear on the poster

Critical words are underlined for illustration

Six presentation lists were created, with each list containing thirty experimental items in each of the six conditions. The one hundred and eighty experimental items in each list were interspersed randomly among ninety unrelated filler sentences to create a single random order and each participant only saw each target sentence once, in one of the six conditions. Four participants from each group were randomly assigned to read each list.

In addition, to obtain a comparative measure of Theory of Mind (ToM) ability across groups, participants completed the Animations Task, based on Abell et al. (2000), in which they watched a series of silent video clips and had to describe interactions between a large red triangle and a small blue triangle. Four clips were designed to prompt an explanation of the triangles’ behaviour in terms of epistemic mental states, such as beliefs, intentions, and deception. Each clip was presented to participants on a computer screen. After the clip was finished, participants described what had happened in the clip. An audio recording of participants’ responses was made for later transcription.

Finally, participants completed a computerised version of the Wisconsin Card Sorting Task (WCST; Grant & Berg, 1948) as a measure of cognitive flexibility. Participants were asked to sort cards according to one of three classification rules: colour (red, blue, yellow, or green), shape (crosses, circles, triangles, or stars), or number of symbols (one, two, three, or four). A series of four cards appeared on the top of the screen which differed in colour, shape, or number of symbols, and one card appeared at the centre bottom. Participants had to figure out which of the three possible sorting rules to adopt according to the feedback that they received after choosing a card. Participants were told that the sorting rule would change throughout the task. There was no practice block, and the experimental block consisted of 128 cards. After clicking on a card, feedback was displayed on the screen stating whether the card had been sorted correctly or incorrectly. If incorrect feedback was received, participants had to switch to a different rule until they received correct feedback. After ten consecutive correct trials, the rule changed. The dependent variable was the total number of perseverative errors*,* defined as the number of times in which participants persisted with an incorrect sorting rule.

### Procedure

Participants were informed about the EEG procedure and experimental task. After electrode application they were seated in a booth where they read the materials from a computer screen (presented using E-Prime software). There were four practice trials to familiarize participants with the procedure, after which the experimenter answered any questions. Each trial began with the presentation of a single centrally-located red fixation cross for 500 ms to signal the start of a new trial. After this time, a white fixation cross appeared for 500 ms. Next, the context sentence was presented on the screen, and participants were instructed to read this sentence and press spacebar on a keyboard to continue when ready. A blank screen appeared for 500 ms, followed by a fixation cross (500 ms). The target sentence was then presented word-by-word, with each word appearing at the centre of the screen for 300 ms, with a 200 ms blank-screen interval between words. A 2500 ms blank-screen interval followed each item. There was no secondary task. Trials appeared in ten blocks of twenty-seven trials. Each block was separated by a break, the duration of which was determined by the participant. Thus, participants were tested in a single session that lasted approximately one hour, during which they were seated in a comfortable chair located in an isolated room. The WASI, ADOS, AQ, animations task and WCST were conducted in a separate testing session.

### Electrophysiological Measures

A Brain Vision Quickamp amplifier system was used with an ActiCap cap for continuous recording of electroencephalographic (EEG) activity from 30 active Ag/AgCl electrodes over midline electrodes Fz, Cz, Pz, and Oz, over the left hemisphere from electrodes Fp1, F3, F7, FC1, FC5, C3, T7, CP1, CP5, TP9, P3, P7, O1, and from the homologue electrodes over the right hemisphere, configured according to the international 10–20 system. EEG and EOG recordings were sampled at 500 Hz with a notch filter at 50 Hz, and electrode impedance was kept below 10 kΩ. Off-line, all EEG channels were recalculated to an average mastoid reference.

Prior to segmentation, EEG and EOG activity was band-pass filtered (0.01–30 Hz, 12 dB/oct). Data was visually inspected for noisy sections or channels, and for other general artifacts, and EEG activity containing blinks was corrected using a semi-automatic ocular ICA correction approach (Brain Vision Analyzer 2.2.1). The continuous EEG record was then segmented into epochs of 1200 ms, starting 200 ms prior to the onset of the target word. Thus, the post-stimulus epoch lasted for a total duration of 1000 ms. Semi-automatic artifact detection software (Brain Vision Analyzer 2) was run, to identify and discard trials with non-ocular artifacts (drifts, channel blockings, EEG activity exceeding ± 50 μV). This procedure resulted in an average trial-loss of 3.69% per condition; average number of trials retained for each group/condition is shown in Table 3.

**Table 3:** Average number of segments retained for ERP analysis by group and condition (M (SD))

	Autistic	TD
Factual consistent	28.33 (2.53)	29.46 (1.06)
Factual inconsistent	28.33 (1.97)	29.46 (0.72)
Counterfactual-counterfactual consistent	28.54 (2.11)	29.25 (1.15)
Counterfactual-counterfactual inconsistent	28.46 (1.98)	29.21 (1.91)
Counterfactual-factual consistent	28.87 (1.51)	29.00 (1.47)
Counterfactual-factual inconsistent	28.75 (1.67)	29.04 (1.92)

### ERP Data Analysis

Procedures for the analysis of EEG data replicated those used in Ferguson and Cane (2015). First, the signal at each electrode site was averaged separately for each experimental condition, time-locked to the onset of the target word, and aligned to a 200 ms pre-target baseline. Mean ERP amplitude was determined in two time intervals relative to target word onset: an early N400 window between 300 and 400 ms and a late N400 window between 400 and 500 ms.

ERP amplitudes were analysed using four regions of interest (ROIs). Lateral electrodes were divided along a left–right dimension, and an anterior–posterior dimension. The two ROIs over the left hemisphere were: left-anterior (Fp1, F7, F3, FC5, FC1), and left-posterior (CP5, CP1, P7, P3, O1); two homologue ROIs were defined for the right hemisphere. ERP amplitudes over midline electrodes (Fz, Cz, Pz), where the N400 is maximal, were analysed separately from data recorded over lateral electrode sites.

Statistical analysis of the N400 was conducted using IBM SPSS software. An ANOVA was performed over lateral electrodes with variables group (autistic *vs.* TD), context (factual *vs.* counterfactual-counterfactual *vs.* counterfactual-factual), consistency (consistent *vs.* inconsistent), hemisphere (left *vs.* right), and ant-pos (anterior *vs.* posterior). ERP amplitudes over midline electrodes were analysed using a group (autistic *vs.* TD) × context (factual *vs.* counterfactual-counterfactual *vs.* counterfactual-factual) × consistency (consistent *vs.* inconsistent) x electrode (Fz, Cz, Pz) ANOVA. Where sphericity was violated, we report the Greenhouse–Geisser corrected effects.

## Results

**Fig. 1:**
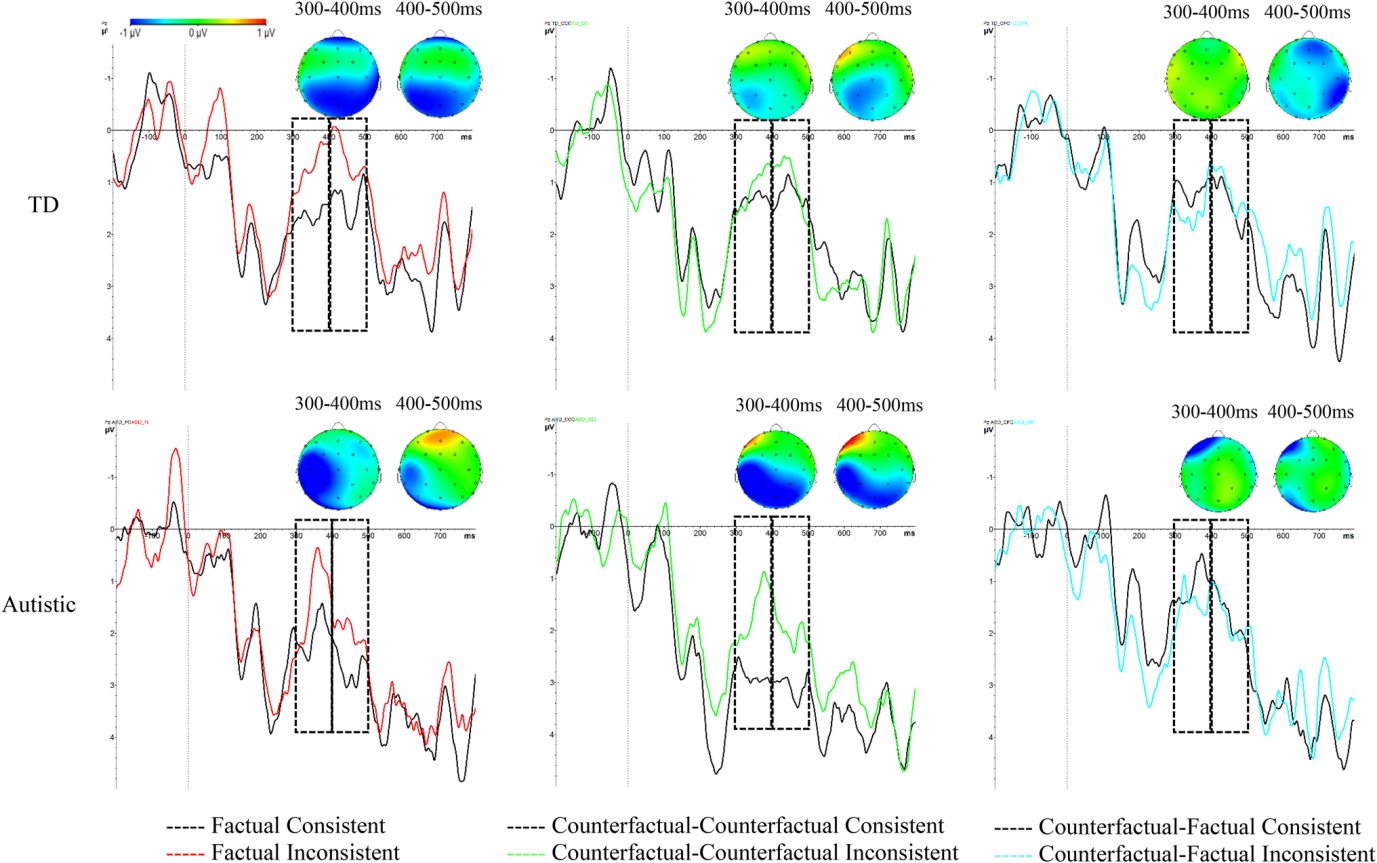
Grand average ERPs elicited by critical words across Factual, Counterfactual-Counterfactual, and Counterfactual-Factual conditions, for TD and autistic groups (dashed rectangles show the time windows used for early (300–400 ms) and late (400–500 ms) N400 analysis). Topographic maps show waveform differences for early (300–400 ms) and late (400–500 ms) ERP windows

For transparency, the full dataset for this experiment is available on the Open Science Framework (OSF) web pages (see https://osf.io/ms5cy/). Grand average ERP waveforms are presented for each context and group in Fig. 1, and mean amplitudes for each time window, group and condition are shown in Tables 4 (lateral) and 5 (midline).

**Table 4:** Mean N400 amplitudes for each time window, group and condition over lateral electrodes (M (SE))

				300–400 ms		400–500 ms	
				Autistic	TD	Autistic	TD
Anterior	Left hemisphere	Factual	Consistent	1.22 (0.37)	0.94 (0.37)	1.75 (0.39)	1.25 (0.39)
			Inconsistent	0.76 (0.42)	0.84 (0.42)	1.65 (0.51)	1.1 (0.51)
		Counterfactual-counterfactual	Consistent	1.68 (0.42)	0.67 (0.42)	1.7 (0.4)	1.21 (0.4)
			Inconsistent	1.66 (0.47)	0.76 (0.47)	1.97 (0.51)	1.47 (0.51)
		Counterfactual-factual	Consistent	1.36 (0.51)	0.93 (0.51)	1.82 (0.5)	1.75 (0.5)
			Inconsistent	0.66 (0.48)	1.14 (0.48)	1.26 (0.56)	1.52 (0.56)
	Right hemisphere	Factual	Consistent	1.31 (0.42)	1.27 (0.42)	1.73 (0.45)	1.08 (0.45)
			Inconsistent	1.12 (0.37)	0.86 (0.37)	1.87 (0.42)	0.87 (0.42)
		Counterfactual-counterfactual	Consistent	1.94 (0.44)	0.83 (0.44)	1.86 (0.44)	1.02 (0.44)
			Inconsistent	1.87 (0.5)	0.96 (0.5)	1.84 (0.51)	1.06 (0.51)
		Counterfactual-factual	Consistent	1.07 (0.48)	0.95 (0.48)	1.3 (0.46)	1.42 (0.46)
			Inconsistent	0.83 (0.41)	1.29 (0.41)	1.18 (0.48)	1.07 (0.48)
Posterior	Left hemisphere	Factual	Consistent	1.76 (0.37)	1.3 (0.37)	2.18 (0.46)	1.24 (0.46)
			Inconsistent	0.62 (0.33)	0.63 (0.33)	1.25 (0.41)	0.45 (0.41)
		Counterfactual-counterfactual	Consistent	2.03 (0.44)	1.05 (0.44)	2.12 (0.43)	1.13 (0.43)
			Inconsistent	1.21 (0.43)	0.64 (0.43)	1.57 (0.46)	0.64 (0.46)
		Counterfactual-factual	Consistent	1.13 (0.46)	0.97 (0.46)	1.63 (0.48)	1.39 (0.48)
			Inconsistent	0.82 (0.42)	1.2 (0.42)	0.98 (0.42)	0.98 (0.42)
	Right hemisphere	Factual	Consistent	1.73 (0.38)	1.62 (0.38)	1.87 (0.47)	1.18 (0.47)
			Inconsistent	1.24 (0.36)	0.67 (0.36)	1.5 (0.39)	0.5 (0.39)
		Counterfactual-counterfactual	Consistent	2.25 (0.48)	1.21 (0.48)	2.04 (0.45)	1 (0.45)
			Inconsistent	1.62 (0.41)	0.89 (0.41)	1.37 (0.44)	0.67 (0.44)
		Counterfactual-factual	Consistent	1.15 (0.42)	1.05 (0.42)	1.2 (0.44)	1.25 (0.44)
			Inconsistent	1.06 (0.42)	1.16 (0.42)	1.05 (0.47)	0.65 (0.47)

**Table 5:** Mean N400 amplitudes for each time window, group and condition over midline electrodes (M (SE))

			300–400 ms		400–500 ms	
			Autistic	TD	Autistic	TD
Fz	Factual	Consistent	1.92 (0.52)	1.59 (0.52)	2.41 (0.57)	1.37 (0.57)
		Inconsistent	1.74 (0.59)	1.14 (0.59)	2.9 (0.67)	1.32 (0.67)
	Counterfactual-counterfactual	Consistent	2.44 (0.59)	1 (0.59)	2.35 (0.6)	1.49 (0.6)
		Inconsistent	2.45 (0.62)	1.44 (0.62)	2.61 (0.71)	1.93 (0.71)
	Counterfactual-factual	Consistent	1.53 (0.62)	1.57 (0.62)	2.02 (0.59)	2.32 (0.59)
		Inconsistent	1.35 (0.5)	1.42 (0.5)	2.16 (0.54)	1.53 (0.54)
Cz	Factual	Consistent	1.92 (0.55)	1.74 (0.55)	2.43 (0.59)	1.46 (0.59)
		Inconsistent	1.38 (0.53)	1.12 (0.53)	2.34 (0.61)	1.01 (0.61)
	Counterfactual-counterfactual	Consistent	2.64 (0.61)	1.43 (0.61)	2.51 (0.6)	1.61 (0.6)
		Inconsistent	2.03 (0.61)	1.16 (0.61)	2.17 (0.63)	1 (0.63)
	Counterfactual-factual	Consistent	1.15 (0.65)	1.44 (0.65)	1.62 (0.61)	1.84 (0.61)
		Inconsistent	1.38 (0.52)	1.48 (0.52)	1.86 (0.57)	1.22 (0.57)
Pz	Factual	Consistent	2.01 (0.45)	1.63 (0.45)	2.67 (0.53)	1.38 (0.53)
		Inconsistent	1.27 (0.49)	0.69 (0.49)	1.97 (0.51)	0.48 (0.51)
	Counterfactual-counterfactual	Consistent	2.87 (0.58)	1.33 (0.58)	3.08 (0.62)	1.23 (0.62)
		Inconsistent	1.6 (0.58)	1.08 (0.58)	1.98 (0.61)	0.93 (0.61)
	Counterfactual-factual	Consistent	1.06 (0.56)	1.15 (0.56)	1.63 (0.57)	1.38 (0.57)
		Inconsistent	1.4 (0.53)	1.55 (0.53)	1.7 (0.55)	1.13 (0.55)

### Animations Task

To verify that ToM competency was compromised in our autistic sample, each verbal transcription was scored on a scale of 0–2 for accuracy (including reference to specific mental states), based on the criteria outlined in Abell et al. (2000). This resulted in a total score for each participant between 0 and 8. Twenty percent of transcripts were scored by two independent raters. Inter-rater reliability across all clips was excellent according to Cicchetti’s (1994) criteria (intra-class correlation = 0.85). Results^2^ showed that autistic participants were significantly impaired at describing the animations in terms of their mental states compared to TD participants (*Ms* = 4.37 *vs.* 5.80 respectively; *t*(42) = 2.22, *p* = 0.032, *d* = 0.68).

### Wisconsin Card Sorting Task

Group differences in participants’ cognitive flexibility were examined, and results^3^ revealed that autistic participants made significantly more perseverative errors compared to TD participants (*Ms* = 14.29 *vs.* 5.54 respectively; *t*(41) = 3.28, *p* = 0.002, *d* = 0.95), suggesting that the autistic group was impaired at switching away from an outdated sorting rule relative to the TD group.

### Early N400 Analyses (300–400 ms)

Analysis of the N400 amplitude in the 300–400 ms time interval over lateral electrodes revealed a significant interaction between ant-pos and consistency, *F*(1, 46) = 8.65, *p* = 0.005, ${\eta }_{p}^{2}$ = 0.16, showing that inconsistent words elicited a significantly more negative-going N400 compared to consistent words over posterior electrodes, *t*(47) = 3.01, *p* = 0.004, but did not differ over anterior electrodes, *t*(47) = 0.69, *p* = 0.491. More importantly, the three-way interactions between electrode, context and consistency over the midline electrodes, *F*(2.9, 131.5) = 4.51, *p* = 0.005, ${\eta }_{p}^{2}$ = 0.09, and between ant-pos, context and consistency over lateral electrodes, *F*(2, 92) = 3.18, *p* = 0.046, ${\eta }_{p}^{2}$ = 0.07, were significant.

Follow-up analyses over the midline revealed that the context x consistency interaction was only marginally significant at electrode Pz, *F*(1.7, 81.0) = 3.09, *p* = 0.058, ${\eta }_{p}^{2}$= 0.06. This interaction was further examined by comparing effects of consistency for each context level. A clear effect of consistency was found for both factual contexts (*t*(47) = 2.51, *p* = 0.02; 0.99 *vs*. 1.83 μV) and counterfactual-counterfactual contexts (*t*(47) = 1.98, *p* = 0.05; 1.34 *vs*. 2.10 μV), revealing the expected increased N400 amplitude following a contextually inconsistent target word compared to a consistent target word. However, no significant difference was found between inconsistent and consistent conditions following a counterfactual-factual context (*t*(47) = 0.98, *p* = 0.33; 1.48 *vs*. 1.11 μV). Follow-up analyses over lateral electrodes did not reveal significant effects over anterior sites (all *F*s < 0.8), however posterior sites showed a main effect of consistency, *F*(1, 47) = 9.08, *p* = 0.004, ${\eta }_{p}^{2}$ = 0.16, and a non-significant context x consistency interaction, *F*(2, 94) = 2.09, *p* = 0.13, ${\eta }_{p}^{2}$ = 0.04. Given the presence of this interaction over midline electrodes and in previous work (Ferguson & Cane, 2015), we conducted follow-up analyses to test the consistency effect at each context level over the posterior sites. Analyses revealed a significant consistency effect within a factual context (*t*(47) = 3.22, *p* = 0.002; 0.80 *vs*. 1.61 μV), a trend within a counterfactual-counterfactual context (*t*(47) = 1.77, *p* = 0.08; 1.10 *vs*. 1.64 μV), and no difference within a counterfactual-factual context (*t*(47) = 0.06, *p* = 0.95; 1.06 *vs*. 1.08 μV).

**Fig. 2:**
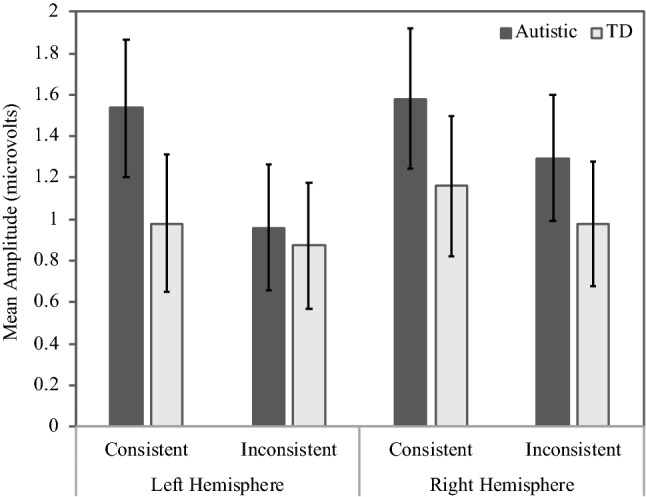
Average ERP response in the early N400 time window (300–400 ms), illustrating the significant group * consistency * hemisphere interaction (error bars show standard errors)

In addition, the lateral electrode analysis revealed a significant group * consistency * hemisphere interaction, *F*(1, 46) = 8.58, *p* = 0.005, ${\eta }_{p}^{2}$ = 0.16. Follow-up analyses showed that the hemisphere * consistency interaction was only significant in the autistic group, *F*(1,23) = 9.37, *p* = 0.006, = ${\eta }_{p}^{2}$0.29, reflecting a significant effect of consistency (inconsistent < consistent) in the left hemisphere, *t*(23) = 2.61, *p* = 0.02, but not in the right hemisphere, *t*(23) = 1.24, *p* = 0.23, in this autistic group (see Fig. 2).

Group did not modulate any other condition effects (all *F*s < 1.5).

### Late N400 Analyses (400–500 ms)

Analysis of the N400 amplitude in the 400–500 ms time interval revealed an interaction between electrode and consistency over midline electrodes, *F*(1.5, 69.7) = 6.61, *p* = 0.005, = ${\eta }_{p}^{2}$0.13, and between ant-pos and consistency over lateral electrodes, *F*(1, 46) = 13.11, *p* = 0.001, ${\eta }_{p}^{2}$ = 0.22. These effects showed that the N400 was more negative-going for inconsistent than consistent critical words over posterior electrode sites (*t*(47) = 3.22, *p* = 0.002; 0.97 *vs*. 1.53 μV), but did not differ over anterior sites (*t*(47) = 0.41, *p* = 0.069; 1.41 *vs*. 1.50 μV). Over the midline, this consistency effect was only significant at electrode Pz, *t*(47) = 2.56, *p* = 0.01.

In addition, the three-way interaction between electrode, context and consistency over midline sites was significant, *F*(3.0, 138.4) = 3.90, *p* = 0.01, ${\eta }_{p}^{2}$= 0.08. Post-hoc analyses did not find a significant context x consistency interaction on any electrode, however analysis on Pz where the N400 was maximal showed a significant consistency effect within a factual context (*t*(47) = 2.09, *p* = 0.042; 1.23 *vs*. 2.03 μV), a trend within a counterfactual-counterfactual context (*t*(47) = 1.73, *p* = 0.09; 1.46 *vs*. 2.16 μV), and no difference within a counterfactual-factual context (*t*(47) = 0.23, *p* = 0.82; 1.42 *vs*. 1.51 μV).

Group did not modulate any condition effects (*F*s < 1.8).

## General Discussion

The present study examined the comprehension of counterfactuals in a sample of autistic adults and their TD peers. Previous work suggests that autistic adults are not disadvantaged in this regard (Black et al., 2018; Ferguson et al., 2019), but these studies are limited by their reliance on single sentence stimuli that maintained just one version of the world (i.e. either factual or counterfactual). Working with such stimuli tests counterfactual comprehension under simplified conditions. In real life, counterfactuals are embedded within complex discourse switching frequently between the real and counterfactual world. In contrast to previous studies, the current experiment utilised an ERP reading anomaly detection task by Ferguson and Cane (2015), in which respondents needed integrate the meaning of two subsequent sentences. Thus, we investigated the processing of counterfactuals under more demanding and realistic circumstances, which facilitated the detection of possible restrictions for autistic adults who, at a group-level, have lower executive capacities than their TD peers.

We predicted that all participants would show appropriate N400 responses when either the factual or counterfactual world was maintained across the two sentences (i.e. a more negative-going wave for contextually inconsistent *versus* consistent critical words), but speculated that the onset of the N400 effect in these conditions might be delayed for autistic compared with TD participants due to general difficulties integrating information in context. Our results showed that both groups of participants exhibited comparable consistency effects for factual and counterfactual-counterfactual contexts, emerging from the early time window of the N400 component over posterior electrode sites. Thus, no evidence was found for disrupted integration and maintenance of a coherent context in autism.

Regarding the most difficult experimental condition, where participants were required to switch from the described counterfactual world to the implied factual world (i.e. counterfactual-factual condition), we expected to see a reversed consistency effect for autistic participants, indicating that readers were continuing to process words according to constraints from the counterfactual world and had not switched to the factual reference frame (based on previous research, and the group-level impairments seen in cognitive flexibility in this sample). Contradicting this hypothesis, our results revealed that the consistency effect was non-significant in the counterfactual-factual context in both groups in the early N400 time window, and showed an overall appropriate N400 effect in the later N400 time window (more negative-going wave for contextually inconsistent *versus* consistent). Thus, again, processing did not differ between autistic and TD readers, and both groups showed evidence that counterfactual and factual versions of the world were equally available to readers at the earliest stages of integration, and readers subsequently favoured the relevant factual world interpretation.

Taken together, the results showed that all participants were able to successfully detect anomalies in sentences that maintained either a factual or counterfactual world, evidenced by more negative-going N400 ERPs following inconsistent than consistent critical words in these contexts. In addition, all participants initially experienced interference from the preceding counterfactual context when they were required to detect anomalies in a context that switched from a counterfactual to factual world, since consistency effects in the early N400 window were non-significant in this context. By the later period of the N400, however, all participants had successfully switched to favour the factual version of the world, evidenced by an appropriate N400 consistency response over posterior electrodes. This suggests that, despite group-level differences in ToM and cognitive flexibility, both groups of participants were sensitive to the linguistic cues provided in the second sentence, and used these appropriately to either maintain a representation of the factual/counterfactual world, or switch between world representations. The results therefore support dual-representation models of counterfactual processing (Byrne, 2002, 2005; Byrne & Tasso, 1999; Fauconnier, 1994, 1997; Johnson-Laird & Byrne, 2002).

The initial non-significant consistency effect in the counterfactual-factual context represents a partial replication of Ferguson and Cane (2015). These authors identified a robust interaction between context and consistency across multiple sites and time windows, suggesting that readers had not switched to favour the factual world in the later N400 window in the counterfactual-factual context (offline ratings showed they did eventually favour the factual interpretation). In the current study, a clear context by consistency interaction was seen in the early N400 window (300–400 ms post critical word onset) over posterior scalp sites, but by the late N400 window (400–500 ms post critical word onset) appropriate consistency effects emerged and did not differ significantly across context conditions. Thus, the findings regarding interference from information presented counterfactually when switching back to the factual world in the present study were not as strong or long-lasting as those in Ferguson and Cane (2015). It is possible that these differences between studies, despite a methodological replication, reflect differences in overall sample size (N = 30 in Ferguson and Cane *versus* N = 48 in the current study), or participant age (mean age in Ferguson and Cane ~ 21 years *versus* ~ 33 years in the current sample). It is known that the ability to process counterfactuals improves with age throughout childhood (Ferrell et al., 2009), and that, in adulthood, language comprehension and vocabulary do not peak until around the age of 50, with both showing significant improvements between the ages of 20 and 30 (Hartshorne & Germine, 2015). Therefore, it is possible that the comparatively older sample in the present study have superior counterfactual discourse processing skills compared to the young adults in Ferguson and Cane (2015) due to increased experience of this type of discourse processing and complex reasoning. Further research is required that systematically sample participants from different age groups across the life span to draw conclusions with regards to age related improvement in counterfactual processing in adulthood.

The finding that counterfactual processing did not objectively differ between autistic and TD adults is consistent with previous research that has used online methods to assess real-time language understanding (e.g. Au-Yeung et al., 2018; Black et al., 2018, 2019; Ferguson et al., 2019; Howard et al., 2017). Nevertheless, this pattern is remarkable given that ASD is characterised by group-level difficulties with executive functioning including cognitive flexibility and working memory (Habib et al., 2019; Williams & Jarrold, 2013), and updating mental representations of language (Peleg et al., 2018)- two processes highly relevant to comprehending counterfactuals. Group-level impairments in cognitive flexibility were observed in the current autistic sample. The anomaly detection task employed here increased demands on respondents’ executive functioning due to the stimuli spanning two sentences (thus requiring coherence of information over a longer narrative) and the need to switch between the counterfactual to the factual world in one condition. Indeed, in Ferguson and Cane (2015; Experiment 2), anomaly detection effects were reduced/absent among participants with low working memory capacity. The fact that counterfactual understanding was unimpaired despite the increased cognitive load is likely to reflect the heterogeneous nature of the autistic phenotype. Despite the group-level impairments in ToM and cognitive flexibility found here, difficulties with executive functioning, contextual integration and meta-learning are not present in every autistic individual. The current findings therefore provide further evidence that counterfactual thinking is not globally impaired among autistic adults and suggests that autistic adults are able to maintain coherence of complex information in ways that would not be predicted by the WCC theory (Booth & Happé, 2010; Frith, 1989; Martin, & McDonald, 2003), the ‘predictive coding theory of autism’ (Van Boxtel & Lu, 2013; Van de Cruys et al., 2014), or the CIP theory (Minshew & Goldstein, 1998; Minshew et al., 1997, 2008; Williams et al., 2015).

Nevertheless, we acknowledge that the methodological approach taken in the current paper (i.e. implicitly assessing understanding in real-time, using ERPs) differs from the response-focused tasks that have been used in most existing research on executive functioning or context integration/prediction in autism (cf. Barzy et al., 2020). It is therefore possible that autistic adults are unimpaired when task requirements are implicit and responses can be measured unobtrusively, but experience difficulties with more deliberate processing that might be subject to response biases. It is also possible that we simply did not have sufficient power to accurately detect these higher-order interaction effects with group in our experiment (our study had an estimated power of 77.3% for the 3-way interaction). However, none of the interactions between group and condition effects even approached significance, and examination of the waveforms and topographies in Fig. 1 shows clear and consistent N400 deflections in the autistic group, supporting our interpretation that appropriate anomaly detection responses were activated online in this group. An important potential limitation of the current study is that our autistic sample was relatively high-functioning, with IQ scores well within the normal range, which may limit the generalisability of our results across the entire spectrum of ASD. Further research is needed to explore whether brain activity and counterfactual comprehension is disrupted in autistic adults with more pronounced cognitive impairments, using Bayesian analyses to formally test the lack of group difference.

Finally, we note that the only group difference that emerged in the present study was in the topographical distribution of the N400 response; the early N400 effect was left lateralised in the autistic group, but bilateral in the TD group. The present experiment did not seek to localise the N400, however this finding raises the possibility that autistic adults activate different neural generators relative to TD adults when processing pragmatic language (see Tesink et al., 2009). For example, previous research has shown that autistic individuals recruit greater resources from the visual cortex when processing language, specifically the left lingual gyrus (Herringshaw et al., 2016). Alternatively, the distinct topographical distribution between groups could reflect the activation of compensatory mechanisms that support processing in autism (for a recent review, see Livingston & Happé, 2017). Formal source localisation analyses using a denser array of electrodes, as well as systematic testing of strategies used to aid comprehension, are needed to investigate these topographical differences in counterfactual discourse processing in autism.

In conclusion, the present study supports dual representation accounts of counterfactual processing, whereby counterfactuals activate representations of both the hypothetical and factual alternatives. Importantly, our results provide further evidence that autistic adults possess an intact ability to track evolving linguistic input in real-time, and use this appropriately to maintain and/or update mental representations of counterfactual and factual events. This finding is surprising given group-level difficulties with executive functioning (evidenced in our sample by impaired cognitive flexibility), contextual integration, and meta-learning in ASD. Thus, given the complex language processing involved in the present study, we conclude that autistic adults are not impaired in comprehending counterfactuals, even when demands on cognitive effort are high.

## Footnotes

1 We acknowledge recent debates about the terminology used to describe autism, and in this paper adopt the identity-first language recommended by autistic adults and parents in Kenny et al. (2016).

2 Note: Four participants from the TD group did not return to complete the animations task, thus data for this task are reported for 24 autistic participants and 20 TD participants.

3 Note: One participant from the autistic group and four participants from the TD group did not return to complete the WCST, thus data for this task are reported for 23 autistic participants and 20 TD participants.
